# Supplementary material for: Structure–function relation of the developing calyx of Held synapse in vivo
Source: J Physiol. 2020 Aug 6;598(20):4603–19. doi: 10.1113/JP279976 (PMC7689866; doi:10.1113/JP279976)
Supplement: Supplementary file 1 — Statistical Summary Document. [file TJP-598-4603-s001.docx]

**Manuscript Title: Structure-function relation for the developing calyx of Held synapse *in vivo***

**Authors:** Sierksma MC, Slotman JA, Houtsmuller AB, Borst JGG

**Animal model used, if applicable:** Wistar rat, pups of undetermined sex, ages of 2 to 8 postnatal days.

**Underlying hypothesis:** Structural and functional parameters of synaptic transmission are intrinsically linked during development, and will thus be correlated.

**Definitions of ‘n’:**

Experimental question 1. Wistar rat pups (undetermined sex).
2-5. MNTB cell.
6. Pair of strongest and second strongest input for a MNTB cell.
8-15. Strong input (>10 V/s) of a MNTB cell (1 input per cell).
16. MNTB cell.
17-22. Strong input (>10 V/s) of a MNTB cell (1 input per cell).
23. MNTB cell.
24-25. Largest or second largest VGluT cluster on a MNTB cell.
26. Pair of largest VGluT cluster and prespike-associated input for a MNTB cell (1 pair per cell).
27. Pair of largest VGluT cluster and strongest input for a MNTB cell (1 pair per cell).
28. Pair of second largest VGluT cluster and second strongest input for a MNTB cell (1 pair per cell).
29. Pair of largest VGluT cluster and competition index for a MNTB cell (1 pair per cell).

**Statistical summary table:**

| **Experimental question number*** | **Finding/ conclusion** | **Experimental location/ variable** | **Mean value** | **SD** | **n** | **P**** | **Units** | **Data comparisons**  **e.g. WT vs KO** | **Statistical test** | **Any other variable** | **Figure in which data are presented** | **Comments**  **e.g. observation** |
| --- | --- | --- | --- | --- | --- | --- | --- | --- | --- | --- | --- | --- |
| 1. current needed for field potential in vivo? | Current activation threshold *in vivo* | MNTB field potential elicited by current stimulation | 83 | 19 | 30 | - | μA | - | - | P2-8 | - | observation |
| 2. For how many cells was the evoked response able to reach the AP threshold? | For most cells | MNTB neurons | 16 | - | 22 | - | cells | - | - | P2-8 | - | observation |
| 3. what is the latency of the evoked inputs? | Two populations of latencies | Latency of responses for MNTB neurons | 1-3 | - | 22 | - | ms | - | - | P2-8 | Figure 1E | observation |
|  |  |  | 4-8 | - |  |  |  |  |  |  |  |  |
|  | Strongest inputs have a shorter latency |  | 1-3 | - | 22 | - | ms | - | - | P2-8 | Figure 1E | observation |
| 4. How many inputs per cell? | We defined 5.6 inputs per cell. | Inputs for MNTB neurons | 5.6 | 1.6 | 32 | - | Inputs | - | - | P2-8 | Figure 2B | observation |
| 5. Change in competition index during development? | Developmental decrease in competition index, because of increase in strength of the strongest input without a developmental change in strength of the second strongest input | Competition index of MNTB neurons vs age of the pup | -0.07 | 0.02 | 32 | **0.002** | Day^-1^ | Slope of regression | ANOVA | P2-8 | Figure 2C |  |
|  |  | Rate of rise of the strongest input vs age of the pup | 4.2 | 1.1 | 32 | **0.002** | V s^-1^ day^-1^ | Slope of regression | ANOVA | P2-8 | Figure 2D | Bonferroni-corrected |
|  |  | Rate of rise of the second strongest input vs age of the pup | -0.22 | 0.15 | 32 | 0.31 | V s^-1^ day^-1^ | Slope of regression | ANOVA | P2-8 | Figure 2D | Bonferroni-corrected |
| 6. Methodological effect of input strength? | No difference in strength for the two strongest inputs per cell whether they are defined from stimulated responses or from spontaneous events. | EPSP rate of rise of input per MNTB cell: strongest input - stimulated | 19 | 10 | 14 | 0.47 | V s^-1^ | Stimulated Vs spontaneous | Welch’s t-test | P2-8 |  |  |
|  |  | Strongest input- spontaneous | 16 | 12 | 18 |  |  |  |  |  |  |  |
|  |  | Second strongest input - stimulated | 3.6 | 1.2 | 14 | 0.91 | V s^-1^ | Stimulated Vs spontaneous | Welch’s t-test | P2-8 |  |  |
|  |  | second strongest input - spontaneous | 3.7 | 1.2 | 18 |  |  |  |  |  |  |  |
| 7. Current threshold of strong inputs? | Threshold was 0.24 mA | Strong input of MNTB cell | 0.24 | 0.09 | 9 | - | mA | - | - | P2-8 |  | observation |
| 8. Variability in activation of strong inputs? | Reliability was 83 % of the stimulations | Reliability of activation of strong input of MNTB cell | 83 | 12 | 9 | - | % | - | - | P2-8 |  | observation |
| 9. Latency of strong inputs? | Latency of strong inputs was 2.1 ms | Latency of strong inputs of MNTB cell | 2.1 | 0.3 | 9 | - | ms | - | - | P2-8 |  | observation |
| 10. Jitter in latency? | Jitter (SD/mean) was low for strong inputs | Jitter in latency of strong inputs of MNTB cell | 2.3 | 0.9 | 9 | - | % | - | - | P2-8 |  | observation |
| 11. Jitter changes during development? | Latency for strong inputs becomes less variable during development. | MNTB input latency coefficient of variation (sd/mean) vs age of the pup | -0.7 | 0.2 | 9 | **0.02** | % day^-1^ | Slope of regression | ANOVA | P2-8 |  |  |
| 12. Latency changes during development? | Latency for strong inputs decreases during development | EPSP latency of MNTB input vs age of the pup | -0.18 | 0.07 | 9 | **0.05** | ms day^-1^ | Slope of regression | ANOVA | P2-8 |  |  |
| 13. Conduction speed at different ages? | We calculated minimal conduction speeds for stimulated, strong inputs | P2-3 | 0.5 | 0.1 | 3 | - | m s^-1^ |  |  |  |  | observation |
|  |  | P4-5 | 0.6 | 0.1 | 5 | - | m s^-1^ |  |  |  |  |  |
|  |  | P6-8 | 0.8 | 0.2 | 2 | - | m s^-1^ |  |  |  |  |  |
| 14. Association of strong inputs with a prespike changes during development? | During development strong inputs become more often associated with a prespike | Association of strong MNTB inputs with a prespike vs age of the pup | 0.2 | 0.06 | 20 | **0.004** | Day^-1^ | Slope of regression | ANOVA | P2-8 | Figure 3B |  |
| 15. Correlation between membrane resistance and the presence of a prespike-associated input? | Membrane resistance of the MNTB cell is correlated with the presence of a prespike-associated input | Presence of prespike | 0.057 | 0.012 | 20 | **1.2 10^-4^** | - | Slopes of regression | ANOVA | P2-8 |  | Membrane resistance was rank-transformed. |
|  |  | Age | 0.083 | 0.060 | 20 | 0.18 |  |  |  |  |  |  |
| 16. Difference in latency for strong inputs with vs w/o prespike? | No difference in EPSP latency of stimulated, strong inputs | EPSP with prespike | 2.1 | 0.4 | 4 | 0.67 | ms | With vs w/o | Welch’s t-test | P2-8 | Figure 3C |  |
|  |  | EPSP w/o prespike | 2.2 | 0.3 | 5 |  |  |  |  |  |  |  |
| 17. Difference in EPSP rate of rise for strong inputs with vs w/o prespike? | No difference in EPSP rate of rise for strong inputs with vs. w/o prespike | Effect of prespike variable | 4.7 | 4.1 | 20 | 0.27 | V s^-1^ | Slopes of regression | ANOVA | P2-8 | Figure 3D |  |
|  |  | Effect of age variable | 2.8 | 1.7 | 20 | 0.12 |  |  |  |  |  |  |
|  |  | EPSP with prespike | 28 | 7 | 10 | - | V s^-1^ |  |  |  |  | Group averages not corrected for age. |
|  |  | EPSP w/o prespike | 19 | 7 | 10 |  |  |  |  |  |  |  |
| 18. What is the coefficient of variation in rate of rise of strong inputs? | Coefficient of variation of EPSP rate of rise (SD/mean) of strong inputs ranges from 7 – 38% | Coefficient of variation of EPSP rate of rise of strong inputs | 22 | 11 | 20 | - | % | - | - | P2-8 |  | observation |
| 19. Difference in EPSP amplitude for strong inputs with vs w/o prespike? | Prespike-associated strong inputs may have a larger EPSP amplitude than the strong inputs without a prespike. | EPSP amplitude of strong inputs with prespike | 19.6 | 3.4 | 10 | 0.058 | mV | With  Vs  w/o | Welch’s t-test | P2-8 |  | No correlation with age was observed for the amplitude, therefore no correction for age.  Absolute EPSP amplitudes are likely underestimated as they often reach AP threshold and become obscured by the AP. |
|  |  | EPSP amplitude of strong inputs w/o prespike | 15.6 | 4.8 | 10 |  |  |  |  |  |  |  |
| 20. Probability of postsynaptic AP following EPSP of strong input? | 75% of EPSPs of strong inputs elicited a postsynaptic AP | Probablity of postsynaptic AP | 75 | 31 | 20 | - | % | - | - | P2-8 |  |  |
| 21. Difference in probability of postsynaptic AP following strong inputs with vs. w/o prespike? | Prespike-associated inputs are more likely to elicit postsynaptic firing than strong inputs without a prespike. | Effect of prespike | 7.0 | 2.7 | 20 | **0.02** | - | Slopes of regression | ANOVA | P2-8 | Figure 3E | Probability of postsynaptic firing is rank-transformed. |
|  |  | Effect of age | 0.4 | 1.2 | 20 | 0.76 | - |  |  |  |  |  |
|  |  | Probability of postsynaptic AP for strong inputs with prespike | 0.88 | 0.22 | 10 | - | - |  |  |  |  | Group averages without correcting for age. |
|  |  | Probability of postsynaptic AP for strong inputs w/o prespike | 0.63 | 0.34 | 10 |  |  |  |  |  |  |  |
| 22. Soma size in 3D reconstructions? | Soma surface is 620 μm^2^ | MNTB neuron reconstruction | 620 | 230 | 20 | - | μm^2^ | - | - | P2-7 | - | observation |
| 23. Contact area of VGluT cluster? | Total somatic area contact by the largest and second largest VGluT cluster per cell | Largest VGluT clusters per MNTB cell | 13 | 16 | 20 | - | μm^2^ | - | - | P2-7 | Figure 5E | observations |
|  |  | Second largest VGluT clusters per MNTB cell | 2.4 | 2.8 | 20 |  |  |  |  |  |  |  |
| 24. Difference in VGluT cluster for cells with vs w/o prespike? | Size of the largest VGluT cluster is larger on cell with a prespike-associated input | Largest VGluT clusters for MNTB cell with a prespike | 28 | 19 | 5 | **0.004** | μm^2^ | With  Vs  w/o | Welch’s t-test | P2-7 |  | Contact area is rank-transformed. |
|  |  | Largest VGluT cluster for MNTB cell w/o prespike | 8 | 11 | 15 |  |  |  |  |  |  |  |
| 25. Correlation between VGluT size and prespike amplitude? | High correlation, but this may be spurious. | Prespike amplitude vs VGluT size | 0.8 | - | 4 | 0.2 |  | Pearson’s *r* | ANOVA | P2-7 |  | observation |
| 26. Correlation between largest VGluT cluster and strongest input? | Size of the largest VGluT cluster predicts strength of strongest input | Contact area of largest VGLuT clusters vs EPSP rate of rise of strongest input; EPSP (V/s) = β area^α^ (α, β) | 0.35 | 0.06 | 20 | **7 10^-5^** | - | α | ANOVA | P2-7 | Figure 5D | Both variables are log-transformed. Reported parameters are not transformed. |
|  |  |  | 6.8 | 0.9 |  |  | V s^-1^ μm^-2α^ | β |  |  |  |  |
| 27. Correlation between second largest VGluT cluster and second strongest input? | Size of the second largest VGluT cluster does not correlate with the strength of the second strongest input | Contact area of second largest VGluT clusters vs EPSP rate of rise of the second strongest input; EPSP (V/s) = β + α area (α, β). | -0.06 | 0.10 | 20 | 0.58 | V s^-1^ μm^-2^ | Slope (α) | ANOVA | P2-7 |  |  |
|  |  |  | 3.8 | 0.4 |  |  | V s^-1^ | Offset (β) |  |  |  |  |
| 28. Correlation between relative contact of the largest VGluT cluster and the competition index? | Relative coverage of largest VGluT clusters on MNTB neuron ranges from 9–98% | Coverage of largest VGluT cluster to total VGluT clusters | 41 | 30 | 20 | - | % |  |  | P2-7 | Figure 5F | Observation |
|  | Competition index is correlated with the relative coverage of the largest VGluT cluster. | Effect of VGluT cluster | -0.69 | 0.17 | 20 | **6.8 10^-4^** | - | Slopes of regression | ANOVA |  |  | Variables were rank-transformed. |
|  |  | Effect of age | 1.6 | 1.0 |  | 0.14 |  |  |  |  |  |  |

*You may use multiple lines for the same question to indicate multiple comparisons

** Authors may wish to make the text bold where p is considered significant against a stated confidence limit
